# Fc-mediated functions of nirsevimab complement direct respiratory syncytial virus neutralization but are not required for optimal prophylactic protection

**DOI:** 10.3389/fimmu.2023.1283120

**Published:** 2023-10-11

**Authors:** Tyler Brady, Corinne Cayatte, Tiffany L. Roe, Scott D. Speer, Hong Ji, LeeAnn Machiesky, Tianhui Zhang, Deidre Wilkins, Kevin M. Tuffy, Elizabeth J. Kelly

**Affiliations:** ^1^ Translational Medicine, Vaccines and Immune Therapies, BioPharmaceuticals R&D, AstraZeneca, Gaithersburg, MD, United States; ^2^ Early Oncology ICA, Oncology R&D, AstraZeneca, Gaithersburg, MD, United States; ^3^ Virology and Vaccine Discovery, Vaccines and Immune Therapies, BioPharmaceuticals R&D, AstraZeneca, Gaithersburg, MD, United States; ^4^ Process and Analytical Sciences, BioPharmaceuticals R&D, AstraZeneca, Gaithersburg, MD, United States; ^5^ Discovery Sciences, BioPharmaceuticals R&D, AstraZeneca, Gaithersburg, MD, United States

**Keywords:** nirsevimab, respiratory syncytial virus, Fc-mediated effector function, anti-RSV F protein monoclonal antibodies, RSV immunoprophylaxis

## Abstract

**Introduction:**

Nirsevimab is an extended half-life (M252Y/S254T/T256E [YTE]-modified) monoclonal antibody to the pre-fusion conformation of the respiratory syncytial virus (RSV) Fusion protein, with established efficacy in preventing RSV-associated lower respiratory tract infection in infants for the duration of a typical RSV season. Previous studies suggest that nirsevimab confers protection via direct virus neutralization. Here we use preclinical models to explore whether fragment crystallizable (Fc)-mediated effector functions contribute to nirsevimab-mediated protection.

**Methods:**

Nirsevimab, MEDI8897* (i.e., nirsevimab without the YTE modification), and MEDI8897*-TM (i.e., MEDI8897* without Fc effector functions) binding to Fc γ receptors (FcγRs) was evaluated using surface plasmon resonance. Antibody-dependent neutrophil phagocytosis (ADNP), antibody-dependent cellular phagocytosis (ADCP), antibody-dependent complement deposition (ADCD), and antibody-dependent cellular cytotoxicity (ADCC) were assessed through *in vitro* and *ex vivo* serological analyses. A cotton rat challenge study was performed with MEDI8897* and MEDI8897*-TM to explore whether Fc effector functions contribute to protection from RSV.

**Results:**

Nirsevimab and MEDI8897* exhibited binding to a range of FcγRs, with expected reductions in FcγR binding affinities observed for MEDI8897*-TM. Nirsevimab exhibited *in vitro* ADNP, ADCP, ADCD, and ADCC activity above background levels, and similar ADNP, ADCP, and ADCD activity to palivizumab. Nirsevimab administration increased *ex vivo* ADNP, ADCP, and ADCD activity in participant serum from the MELODY study (NCT03979313). However, ADCC levels remained similar between nirsevimab and placebo. MEDI8897* and MEDI8897*-TM exhibited similar dose-dependent reduction in lung and nasal turbinate RSV titers in the cotton rat model.

**Conclusion:**

Nirsevimab possesses Fc effector activity comparable with the current standard of care, palivizumab. However, despite possessing the capacity for Fc effector activity, data from RSV challenge experiments illustrate that nirsevimab-mediated protection is primarily dependent on direct virus neutralization.

## 1 Introduction

Respiratory syncytial virus (RSV) is a leading cause of lower respiratory tract infection (LRTI), with seasonal RSV epidemics producing significant global morbidity among infants and children aged <24 months (1–6). The risk of severe RSV disease is highest within the first 12 months of life and ~70% of hospitalizations during this time occur in otherwise healthy infants born at term (3–7). Despite considerable annual burden on public health, the development of pediatric RSV vaccines has been hampered by historical observations of antibody-dependent disease enhancement (ADE) following administration of a formalin-inactivated RSV (FI-RSV) vaccine in the 1960s, and challenges in eliciting sustained anamnestic immune responses from the neonatal immune system (8, 9).

Immunoprophylaxis with monoclonal antibodies (mAbs) can provide neutralizing antibodies independent of the host’s immune system, and thus are uniquely placed to protect infants with developing immune systems against disease (10). The humanized murine anti-RSV Fusion (F) protein mAb, palivizumab, has an established history in preventing hospitalization from RSV LRTI, without any evidence of ADE since its licensure in 1998 (11, 12). However, due to the cost of monthly injections, policymakers have reserved palivizumab use for infants at high risk of severe RSV disease (i.e., infants born pre-term and infants born with congenital heart disease and chronic lung disease of prematurity) (13). This left an unmet need for widely available and cost-effective RSV prophylaxis for the benefit of all infants and spurred the development of nirsevimab – a recombinant human anti-RSV F protein mAb. In late 2022, nirsevimab was approved for the prevention of RSV lower respiratory tract disease in neonates and infants during their first RSV season in the European Union and Great Britain. Subsequently in mid-2023, nirsevimab was approved in Canada and the United States for use in neonates and infants in their first RSV season, and in children up to 24 months of age who remain vulnerable to severe RSV disease through their second RSV season (14–16).

Nirsevimab targets a highly conserved discontinuous neutralizing epitope within site Ø of the pre-fusion confirmation of the F protein, thereby limiting RSV infection by inhibiting viral fusion with the host cell (17). Nirsevimab was derived from a precursor anti-RSV pre-fusion F protein mAb, MEDI8897*, through the addition of the triple amino acid M252Y/S254T/T256E (YTE) substitution to the fragment crystallizable (Fc) region, thereby prolonging *in vivo* serum half-life (mean 71 days) and enabling a single intramuscular injection of nirsevimab to confer protection for an entire RSV season (17–22). Nirsevimab has consistently demonstrated efficacy of 70–80% against medically attended RSV LRTI >150 days post dose in multiple analyses of pre-term and full-term infants from the pivotal Phase 2b (NCT02878330) and Phase 3 MELODY studies (NCT03979313) (18, 19, 23, 24). Additionally, nirsevimab delivered an 83% reduction against hospitalizations from RSV LRTI in preliminary analyses of the real-world Phase 3b HARMONIE study (NCT05437510) (25).

Due to the absence of a well-established correlate of protection, investigational RSV vaccines and mAbs have largely been assessed based on their virus neutralization activity (17, 26–30). However, emerging data from animal models and studies of maternally transferred antibodies suggest that broader Fc effector functions, such as antibody-dependent neutrophil phagocytosis (ADNP), antibody-dependent cellular phagocytosis (ADCP), antibody-dependent complement deposition (ADCD), and antibody-dependent cellular cytotoxicity (ADCC) may be additionally important mediators of RSV disease severity (31–35). Although immunoglobulin 1 (IgG1) antibodies such as palivizumab and nirsevimab are regarded as strong inducers of ADNP, ADCP, ADCD, and ADCC, the specific contributions of Fc effector functions to nirsevimab-mediated protection are not yet established (35, 36). In this context, we have used a combination of preclinical and *ex vivo* models to explore whether Fc-mediated effector functions contribute to nirsevimab-mediated protection.

## 2 Materials and methods

### 2.1 Overview of MEDI8897*-derived antibodies

Nirsevimab was derived from MEDI8897* through the addition of the YTE modification (17). MEDI8897* and MEDI8897*-TM antibodies were used to evaluate the contributions of Fc effector activity in these analyses. MEDI8897* was compared to nirsevimab to infer the influence of the YTE modification on Fc effector activity. The addition of the triple amino acid L234F/L235E/P331S (TM) modification has previously been shown to disrupt antibody-FcγR binding and reduce Fc effector functions (37). MEDI8897*-TM was used as a negative control.

### 2.2 Surface plasmon resonance evaluation of mAb binding to Fc γ receptors

mAb binding to FcγRs was measured with surface plasmon resonance (SPR), a routine technique for characterizing the interaction between Fc receptors and IgGs (38). An assay was conducted to measure the binding affinity of MEDI8897* samples to a panel of FcγRs. The assay was performed on a Biacore™ T200 instrument using Hepes Buffered Saline-EP+ assay running buffer. A mouse anti-His antibody (BioRad, MCA1396) was diluted to 20 μg/mL in 10 mM sodium acetate at pH 5.0, and ~7000 response units (RU) were immobilized to two flow cells of a CM5 sensor chip using a standard amine coupling protocol. One flow cell served as a reference surface and the second flow cell as the experimental surface. During each assay cycle, one FcγR at 1–2 µg/mL was captured to the anti-His antibody on the experimental flow cell. A single concentration of sample was then passed over both flow cells, followed by regeneration of both flow cells. The FcγRs were injected at a flow rate of 10 µL/min for 40 seconds for capture. The test antibodies were then injected at a flow rate of 30 µL/min for a 60-second association time, followed by 60-second dissociation time (120-second association, 120-second dissociation for FcγRI). A two-fold dilution series of the test antibodies was used against all receptors except FcγRI, where a three-fold dilution series was used. The sensor surface was regenerated with one injection of 20 mM hydrochloric acid at 30 µL/min for 40 seconds. Using Biacore™ T200 Evaluation Software, the sensorgram data were adjusted against the reference flow cell and a buffer-only injection control, then fitted to a steady state affinity model for FcγRIIA, FcγRIIB, and FcγRIIIA-158V, or a 1:1 binding model for FcγRI, to generate the affinity constant (K^D^) for the interaction.

### 2.3 Cell lines

The human promyelocytic HL-60, and monocytic THP-1, cell lines were used as model systems to study ADNP and ADCP, respectively. HL-60 cells were cultured in Iscove’s Modified Dulbecco’s Medium (Gibco, 12440053) with 20% heat-inactivated fetal bovine serum (FBS). Flasks were seeded with 1.5 x 10^5^ HL-60 cells/mL. Prior to use in ADNP assays, HL-60 cells underwent granulocytic differentiation into neutrophil-like cells by incubating cultures with 1.3% dimethylsulfoxide for 5 days at 37°C and 5% CO_2_ (39). THP-1 cells were cultured in Roswell Park Memorial Institute (RPMI)-1640 (Gibco, 22400089) with 10% FBS. HEp-2 cells were used as permissive hosts for RSV replication. HEp-2 cells were cultured in Minimal Essential Medium (MEM)/Earle’s Balanced Salt Solution (Gibco, 11095080) with 5% FBS. Jurkat cells engineered to express CD16 (FcγRIIIA-158V) and a nuclear factor of activated T cells (NFAT) luciferase reporter were used as a surrogate for NK cells in the ADCC reporter bioassay (Promega G7010). Jurkat cells were thawed following manufacturer recommendations (37°C water bath for 2–3 minutes, mixed by pipetting twice, then 630 μL of cells were added to 3.6 mL of assay medium). Cells were used immediately in the assay, with RPMI-1640 with 4% low IgG FBS (Promega G7010) used as the ADCC assay medium.

### 2.4 Pre-F trimer protein expression and purification

RSV DS-Cav1 pre-F protein was expressed by transient transfection in Chinese hamster ovary cells using Polyethylenimin (40). The culture supernatants were harvested ~10 days after transfection. The culture supernatants were sterile filtered prior to buffer exchange and concentrated using tangential flow filtration. RSV F glycoproteins were first purified by nickel-affinity chromatography and followed by size-exclusion chromatography.

### 2.5 RSV F protein bead coupling for bead-based assays

A two-step carbodiimide reaction was used to couple recombinant RSV A2 pre-F protein trimer to either yellow-green or blue carboxylate-modified beads (Invitrogen, F8823/F8815). Beads (10 μL) were activated for 30 minutes at room temperature using 80 μL of sodium phosphate buffer (pH 6.2) with 10 μL of 50 mg/mL Sulfo-N-hydroxysulfosuccinimide (Pierce, A39269) and 10 μL of 50 mg/mL ethyl dimethylaminopropyl carbodiimide hydrochloride (Pierce, 22980). Beads were then washed three times with 50 mM 2-(N-morpholino) ethanesulfonic acid (MES), pH 5.0 (Thermo Scientific Chemicals, J62081.AK), by spinning at 16,000 xg for 10 minutes. After activation, 20 μg of RSV pre-F protein was added and incubated at room temperature for 2 hours. Beads were then washed once in phosphate buffered saline (PBS) with 0.1% bovine serum albumin (BSA), 0.02% Tween-20, and 0.05% sodium azide, and centrifuged at 4000 xg for 20 minutes. The coupled beads were blocked with 5% BSA in PBS overnight at 4°C, then washed and resuspended in 1 mL of 0.1% BSA in PBS.

### 2.6 Antibody-dependent neutrophil phagocytosis

ADNP was investigated based on a method by Worley, et al. (41). Briefly, blue-coupled beads (10 µL/well) were added to 96-well u-bottom plates with 10 µL/well of diluted purified mAb or clinical sample and were incubated in the dark for 2 hours at 37°C and 5% CO_2_ to form immune complexes. After incubation, the immune complexes were washed with 0.1% BSA diluted in PBS and spun at 4000 xg for 20 minutes. HL-60 cells were prepared at 5 x 10^5^ cells/mL and 200 μL were added to each well. Following overnight incubation, cells were stained with anti-CD11b (BD 568454) and fixed in 4% paraformaldehyde (PFA) for 15 minutes. Fluorescence was measured on a BD LSRFortessa using the 4′,6-diamidino-2-phenylindole channel. The data were reported as the average (n = 3 technical replicates) phagocytic score, which was calculated using the formula below.


$$phagocytic\;score=\;\frac{percentage\;of\;bead^{+}\;cells\times gMFI\;of\;bead^{+}\;cells\;}{10,000}$$

gMFI, geometric mean fluorescence intensity.

### 2.7 Antibody-dependent cellular phagocytosis

Yellow-green coupled beads and immune complexes were prepared in 96-well u-bottom plates per the same conditions as the ADNP method. THP-1 cells were prepared at a density of 1.25 x 10^5^ cells/mL and 200 μL of cells were added to each well. The next day, plates were centrifuged at 600 xg for 20 minutes, and cells were fixed with 4% PFA for 15 minutes. Following fixation, cells were spun at 600 xg for 20 minutes and then resuspended in 0.1% BSA diluted in PBS. Fluorescence was measured on a BD LSRFortessa using the fluorescein isothiocyanate channel. The data were reported as the average (n = 3 technical replicates) phagocytic score.

### 2.8 Antibody-dependent complement deposition

Immune complexes were formed by adding 10 µL of blue-coupled beads to 96-well u-bottom plates and incubating with 10 µL diluted purified mAb or clinical sample for 2 hours at 37°C and 5% CO_2_. After incubation, the immune complexes were washed with RPMI medium (Gibco, 21870076). Guinea pig serum (Sigma, 234395) was diluted 1:60 in gelatin veronal buffer (Sigma, G6514), added to the plates, and incubated for 50 minutes at 37°C and 5% CO_2_ to facilitate binding between complement proteins and anti-RSV F protein immune complexes. The reaction was then stopped by washing the plates twice with 15 mM ethylenediaminetetraacetic acid in PBS. To detect complement deposition, plates were incubated with fluorescein isothiocyanate-conjugated goat anti-guinea pig complement C3 antibody (MP Biomedicals, 0855385) for 20 minutes in the dark. Plates were washed with PBS and fluorescence was measured on a BD LSRFortessa. The results were reported as the average (n = 3 technical replicates) median fluorescent intensity.

### 2.9 Antibody-dependent cellular cytotoxicity

The cytotoxic activity of Jurkat cells expressing CD16 (FcγRIIIA-158V) and a NFAT luciferase reporter on RSV-infected HEp-2 cells was measured using a bioluminescent ADCC reporter assay (Promega G7010). HEp-2 target cells were prepared by seeding 25,000 cells per well in 96-well plates. Cells were plated with RSV A000, (i.e., a recombinant RSV resembling the contemporary RSV A sequence (42)) at a multiplicity of infection of 1 and incubated for 24 hours at 37°C and 5% CO_2_. Either purified antibody or clinical samples were then added to the plates along with 75,000 Jurkat effector cells per well following manufacturer kit recommendations. Plates were incubated for 6 hours at 37°C and 5% CO_2_. Bio-Glo™ luciferase reagent was reconstituted following manufacturer recommendations (Promega G7010) and then added to the plates. Plates were incubated at RT in the dark for 30 minutes. Luminescence was read on an EnVision plate reader.

### 2.10 Collection of immunized serum

Serum samples were collected from participants during the Phase 3 MELODY study; a placebo-controlled study of nirsevimab in healthy pre-term and term infants (NCT03979313) (19, 24). In the original MELODY protocol, participants provided serum samples for exploratory analyses prior to nirsevimab dosing at Baseline on study Day 1 and Days 31, 151, and 361 (19, 21). The serum samples used in this analysis were collected from participants enrolled at sites in the European Union under a revised protocol (EudraCT 2019-000114-11), which replaced the Day 31 serum collection with an earlier collection on Day 15. Samples were stored at –80°C prior to being pooled for analysis. Baseline and Day 15 serum pools were created using samples from ten unique participants per time point. Three nirsevimab pools (i.e., 30 participants per time point) and one placebo pool (i.e., 10 participants per time point) were created for this analysis.

IgG-depleted serum was used as a negative control to assess background signal from serum in the absence of IgG. IgG-depleted serum was prepared by resuspending lyophilized powder in 0.5 mL of deionized water following manufacturer instructions (Sigma S5143).

### 2.11 RSV microneutralization susceptibility assay

The neutralization potencies of nirsevimab, MEDI8897*, MEDI8897*-TM, and palivizumab were evaluated against recombinant RSV A and RSV B viruses in a microneutralization susceptibility assay. Each mAb was serially diluted in 96-well plates prior to the addition of a fixed concentration of RSV A000 or RSV B000 at a median tissue culture infectious dose (TCID_50_) of 500 per well and was incubated for 1 hour followed by the addition of HEp-2 cells. After 5 days of incubation at 37°C, RSV-infected cells were fixed and stained with anti-RSV mAb (Millipore, Cat# MAB8262 Clone 133-1H at 1:5000 dilution) and a horseradish peroxidase-labelled detection antibody (Dako polyclonal goat anti-mouse IgG-HRP Cat# P0447 at 1:4000 dilution). Tetramethylbenzidine substrate was added and the OD_450nm_ was measured. Half-maximal inhibitory concentrations (IC_50_) were calculated using 4PL nonlinear curve fitting on the GraphPad Prism (version 9.4.0) software suite and compared to the IC_50_ values of reference viruses on the same plate, to determine fold-change in IC_50_ values.

### 2.12 RSV challenge in cotton rats

All procedures were performed in accordance with federal, state, and institutional guidelines in an AAALAC-accredited facility, The MedImmune Institutional Animal Care and Use Committee (IACUC) board approved this research under a specified protocol (MI-16-0014), and all animal work was performed in accordance with the IACUC policies. MedImmune is registered with the U.S. Department of Agriculture (USDA) and applies the standards outlined in the Guide for the Care and Use of Laboratory Animals to its institutional animal care and use program (43). Animals were lightly anesthetized with isoflurane for immunizations and blood draws and were euthanized with CO_2_ for terminal organ harvests.

Sixty 4–6-week-old, female cotton rats (Envigo, Dublin, VA) were housed under pathogen-free conditions at MedImmune (Gaithersburg, MD). Eight rats were used per dosing weight group to ensure reliability of the findings. Weight-based doses (2.0, 1.0, or 0.5 mg/kg) of MEDI8897* or MEDI8897*-TM were administered intramuscularly (n = 8 per dose group for each agent). The anti-human immunodeficiency virus gp120 envelope glycoprotein mAb, R347, was administered at 2.0 mg/kg as a negative control (n = 6), and six rats were used as an untreated control group. Blood was collected 1 day later by retroorbital bleed for quantification of mAb serum concentration. Animals were challenged with RSV A2 diluted in PBS at a dose of 1 x 10^6^ plaque-forming units per rat via intranasal inoculation immediately following blood collection. Animals were euthanized at 4 days post challenge.

### 2.13 Plaque assay

RSV titers in cotton rat lungs and nasal turbinates were determined by plaque assay.

24-well plates were seeded with HEp-2 cells at 2.5 x 10^5^ cells/well (cell culture media comprised Gibco #12360 MEM, 5% heat-inactivated FBS, and 1% PenStrepGlutamine [Gibco 100x #10378-016]) and were incubated overnight at 37°C. Dilutions of thawed lung or nasal turbinate homogenates were prepared (1:10 and 1:100 dilutions) on ice packs or wet ice. All media were removed from each well by aspiration. Cells were then infected by adding 250 μL of each dilution in duplicate to the cell monolayers, and then gently rocked and incubated at 37°C for 1 hour. The viral solution was then removed by aspiration. Methylcellulose was used for the overlay (8%, 4000 cP [Sigma M-0512], autoclave dried and dissolved in HEp-2 media); 1 mL of warm methylcellulose (0.8% in cell culture media) was added to each well, which were then incubated at 37°C for 5 days. After 5 days, the methylcellulose was removed by aspiration and 500 μL of crystal violet fix/stain solution (1 g crystal violet, 200 mL methanol in 800 mL water) was added. After 20 minutes of incubation, the stain was removed and the wells were rinsed twice with water. The wells were patted dry and air dried, after which the plaques were counted.

### 2.14 Statistics

For the *in vitro* analyses, each monoclonal antibody was tested in an 8-point dilution curve for each Fc effector assay. Three technical replicates of each dilution were tested for each antibody, with mean values reported per assay. Statistical analysis was performed with GraphPad Prism (version 9.4.0). Area under the curve (AUC) was measured for each biological replicate. The mean AUC over the lowest background value (± standard deviation [SD]) is reported with an ordinary one-way analysis of variance (ANOVA) with a *post-hoc* Dunnett multiple comparisons test to compare nirsevimab to the control mAbs, palivizumab and R347.

A similar statistical approach was used for the *ex vivo* analyses. Briefly, each serum pool was tested in an 8-point dilution curve for each assay. ADNP was tested in a 6-point dilution curve due to high background levels at the lower end of the assay range. Three technical replicates of each dilution were tested for each serum pool, with mean values reported per assay. AUC was measured for each biological replicate. The nirsevimab Baseline and Day 15 pools were combined for an overall estimate at both time points. The mean AUC over the lowest background value (± SD) is reported. To account for more replicates after combining the three nirsevimab Baseline and Day 15 pools compared with placebo and IgG-depleted serum, a linear mixed-effect model was fitted to log-transformed AUC values. The false discovery rate correction was applied to compare nirsevimab**-**immunized serum at Baseline and on Day 15, nirsevimab-immunized serum on Day 15 and placebo Day 15, and nirsevimab-immunized serum on Day 15 and IgG-depleted serum. Statistical analysis was performed in R (version 4.1.3). To account for the relatedness of the nirsevimab Baseline and Day 15 serum pools, these samples were analyzed as Imer(log(AUC) ~Group + (1|Subj)). (1|Subj) is a random effect that considers the relatedness of nirsevimab replicates within both groups.

Sample size estimation for the cotton rat challenge experiments was completed prior to experimentation using a two-group Satterthwaite T-test with a two-sided significance level of 0.05 with 80% power to detect differences between treatment groups based on pilot data. Sample sizes were estimated using nQuery Advisor+ nTerim3.0. Significance between MEDI8897* and MEDI8897*-TM was determined by one-way ANOVA.

## 3 Results

### 3.1 Comparing Fc γ receptor binding by surface plasmon resonance confirms the relative binding affinities of Fc modifications

The binding activity of nirsevimab, MEDI8897* (i.e., nirsevimab without the YTE modification), MEDI8897*-TM (i.e., MEDI8897* with ablated Fc receptor binding thus impairing effector functions), and palivizumab to a range of FcγRs was assessed using SPR (**Table 1**). Nirsevimab exhibited an 8.3-fold reduction in binding affinity to FcγRI, and a 1.8–2.5-fold reduction in binding affinity to FcγRIIA and FcγRIIIA-158V compared with MEDI8897*, suggesting that the YTE modification led to a modest reduction in FcγR binding affinity. As expected, the binding affinity of MEDI8897*-TM was greatly reduced compared with MEDI8897*, with ~190-fold, ~9-fold, and ~5-fold reductions in binding affinities to FcγRI, FcγRIIA, and FcγRIIIA-158V, respectively. MEDI8897* and palivizumab exhibited similar binding affinities to all FcγRs tested.

**Table 1:** Comparison of anti-RSV F protein Fc γ receptor binding by surface plasmon resonance.

mAb	FcγR binding affinity (nM)			
	FcγRI	FcγRIIA	FcγRIIB	FcγRIIIA-158V
Nirsevimab	10.3	9300	N/A	2110
MEDI8897*	1.24	3720	13,500	1150
MEDI8897*-TM	240	34,400	N/A	5150
Palivizumab	1.01	2780	12,500	882

### 3.2 Nirsevimab and palivizumab display comparable *in vitro* Fc effector function activity

**Figure 1:**
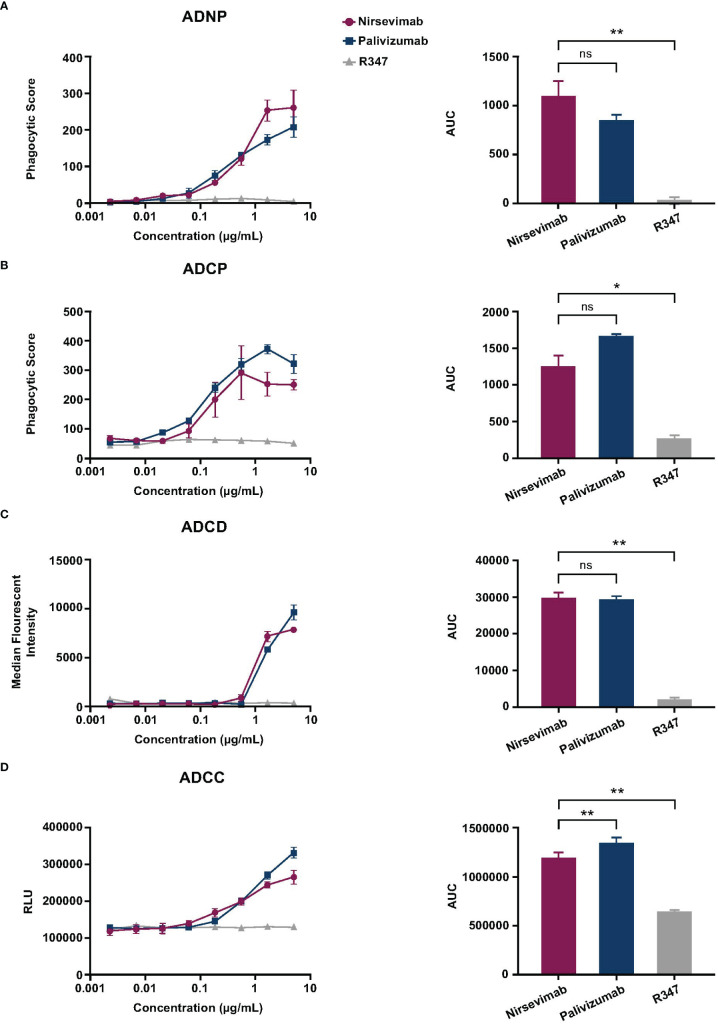
Evaluation of nirsevimab and palivizumab Fc effector activity *in vitro.* Nirsevimab, palivizumab, and R347 (range: 2.29–5000 ng/mL) were evaluated for *in vitro* **(A)** ADNP, **(B)** ADCP, **(C)** ADCD, and **(D)** ADCC activity. Samples were tested in technical triplicates with the **(A, B)** average phagocytic score, **(C)** average complement deposition (measured as median florescence intensity), or **(D)** relative luminescence units (RLUs) shown for each mAb concentration. An AUC analysis of each mAb tested across eight dilutions is shown for each respective assay. The mean AUC value (± SD) is reported with an ordinary one-way ANOVA with *post-hoc* Dunnett multiple comparisons test. Not significant (ns); p>0.05; *p<0.05; **p<0.01.

We next conducted *in vitro* ADNP, ADCP, ADCD, and ADCC assays to compare the Fc effector activities of nirsevimab and palivizumab against R347 as a negative control. Nirsevimab ADNP activity was significantly above R347 background levels (p ≤ 0.01) (**Figure 1A**). Nirsevimab and palivizumab demonstrated comparable levels of ADNP activity (not significant [ns], p>0.05). Nirsevimab ADCP activity was statistically significant (p ≤ 0.05) compared with that of R347 (**Figure 1B**), and was comparable to palivizumab ADCP activity (ns, p>0.05). Nirsevimab ADCD activity was significantly greater than that observed with R347 (p ≤ 0.01), and was once again comparable to palivizumab (ns, p>0.05) (**Figure 1C**). Nirsevimab exhibited ADCC activity that was significantly greater than that observed with R347 (p ≤ 0.01) but showed lower levels of ADCC activity compared with palivizumab (p ≤ 0.01) (**Figure 1D**).

### 3.3 Nirsevimab displays *ex vivo* Fc effector activity in MELODY study participant serum

Nirsevimab *ex vivo* Fc effector activity was assessed in participant serum from the Phase 3 placebo-controlled MELODY study (19). Participant serum samples obtained at either Baseline or on Study Day 15 were pooled and evaluated for ADNP, ADCP, ADCD, and ADCC activity against an IgG-depleted serum control.

**Figure 2:**
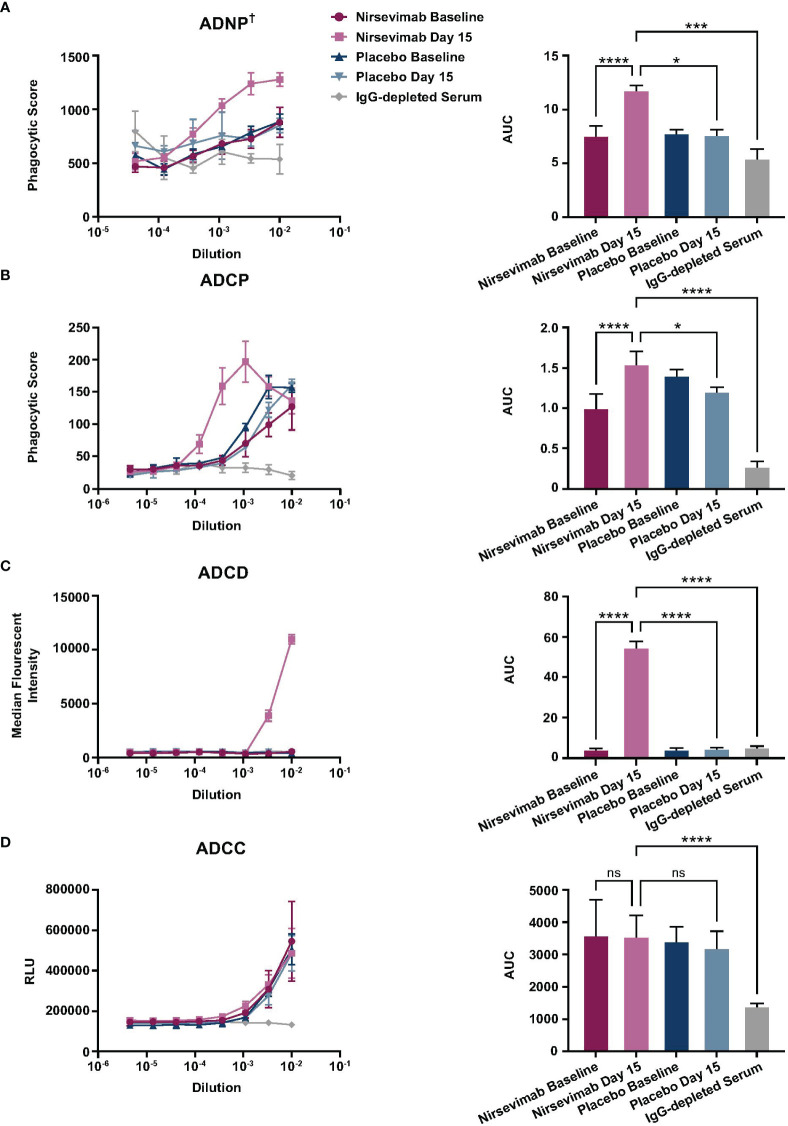
Evaluation of *ex vivo* nirsevimab Fc effector activity in participant serum from the Phase 3 MELODY study. *Ex vivo* nirsevimab **(A)** ADNP^†^, **(B)** ADCP, **(C)** ADCD, and **(D)** ADCC activity was measured using participant serum from the Phase 3 placebo-controlled MELODY study (NCT03979313). Nirsevimab-immunized and placebo participant serum pools were diluted in PBS. Diluted pools were tested in technical triplicates at the dilutions shown (range: 10^–6^–10^–2^) with the **(A, B)** average phagocytic score, **(C)** average complement deposition (measured as median florescence intensity), or **(D)** relative luminescence units (RLUs) for each dilution shown. An AUC analysis for all dilutions within each serum pool is shown for each respective assay. The mean AUC value (± SD) is reported with a linear mixed-effect model of log (AUC) and false discovery rate correction to compare Fc effector activity between nirsevimab-immunized serum on Day 1 and 15; nirsevimab Day 15 and placebo Day 15; and nirsevimab Day 15 and IgG-depleted serum. Three pools of nirsevimab samples were tested at Baseline and at Day 15. For comparison with placebo time points and IgG-depleted serum, nirsevimab sample results were combined into one pool for each time point. The reported SD reflects the technical replicates of the three separate pools. ^†^One replicate of IgG-depleted serum was excluded from the ADNP analysis, as its phagocytic score ranged from 1120–2037, which exceeded the highest scores seen in all the other Fc effector activity groups. Not significant (ns); p>0.05; *p<0.05; ***p<0.001; ****p<0.0001.

A statistically significant increase in ADNP activity was observed in nirsevimab-immunized serum between Baseline and Day 15 (p ≤ 0.0001) (**Figure 2A**), and Day 15 levels were significantly greater than corresponding levels in placebo (p<0.05). ADNP activity in nirsevimab serum on Day 15 was also significantly higher than that observed in IgG-depleted serum (p<0.001). The ADCP activity of nirsevimab-immunized serum increased significantly between Baseline and Day 15 (p ≤ 0.0001) (**Figure 2B**). Similarly, ADCP activity was significant over levels observed in placebo at Day 15 (p<0.05). ADCP activity in nirsevimab-immunized serum on Day 15 was significantly higher than that in IgG-depleted serum (p ≤ 0.0001). Nirsevimab-immunized serum at Day 15 exhibited statistically significantly greater ADCD activity compared with that of nirsevimab at Baseline, placebo at Day 15, and IgG-depleted serum (all p ≤ 0.0001) (**Figure 2C**). Similar levels of ADCC activity were observed between nirsevimab-immunized serum and placebo at Baseline and Day 15 time points (both ns; p>0.05) (**Figure 2D**). However, ADCC levels among nirsevimab recipients were higher than those observed in IgG-depleted serum (p ≤ 0.0001).

### 3.4 Comparing *in vitro* neutralization activity confirms that the YTE and TM modifications do not impact neutralization potency

The neutralization potencies of nirsevimab, MEDI8897*, MEDI8897*-TM, and palivizumab against recombinant RSV A and RSV B viruses were evaluated via an RSV microneutralization susceptibility assay (**Table 2**). Nirsevimab, MEDI8897*, and MEDI8897*-TM exhibited similar neutralization activity against both RSV A and RSV B viruses, illustrating that the YTE and TM modifications do not impact neutralization potency. Palivizumab showed decreased neutralization potency compared with nirsevimab and MEDI8897*, which was consistent with previous analyses (17).

**Table 2:** Comparison of *in vitro* neutralization activity of anti-RSV F protein mAbs.

mAb	IC_50_ (ng/mL)			
	RSV A (rRSV A000)		RSV B (rRSV B000)	
	Mean	SD	Mean	SD
Nirsevimab	5.42	0.03	9.71	0.86
MEDI8897*	6.37	2.01	8.51	0.28
MEDI8897*-TM	4.50	0.00	4.81	0.84
Palivizumab	244.20	53.17	358.15	33.73

### 3.5 Fc effector functions are not required for nirsevimab-mediated protection from RSV infection in cotton rats

A challenge experiment was performed in cotton rats to further evaluate the role of Fc effector function in nirsevimab-mediated protection from RSV. MEDI8897* was used as a surrogate for nirsevimab in these experiments as YTE substitutions have previously been shown to significantly decrease antibody exposure in rodents due to increased affinity for murine neonatal FcγRs (20).

**Figure 3:**
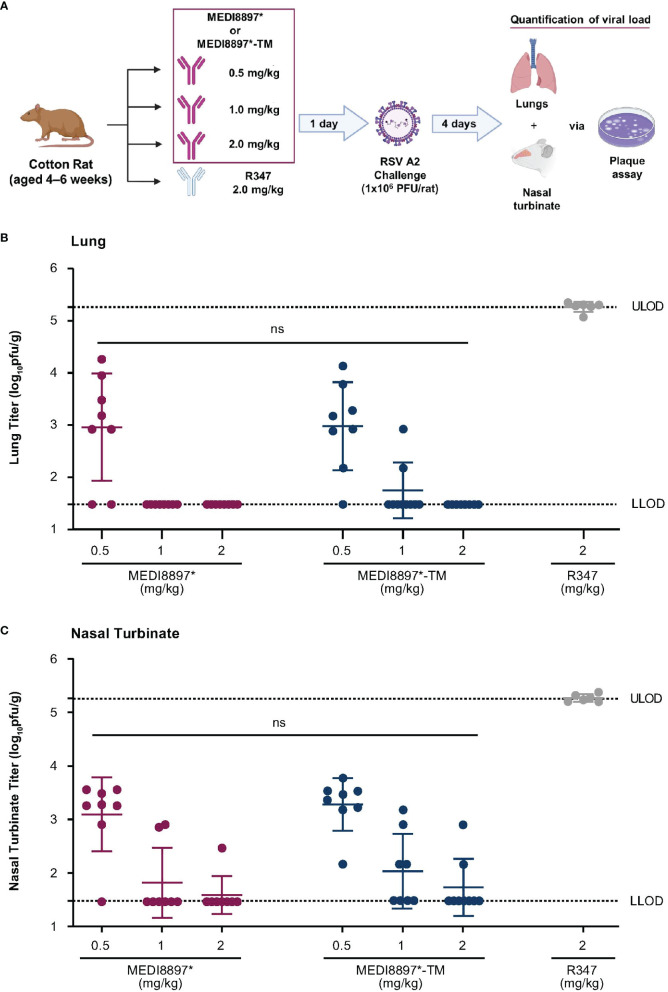
Evaluation of nirsevimab-mediated protection from RSV infection in cotton rats. **(A)** Weight-based doses of MEDI8897*, MEDI8897*-TM, or R347 negative control mAb were administered to cotton rats 1 day prior to challenge with RSV A2 (1 x 10^6^ plaque-forming units [PFU] per rat). Animals were sacrificed 4 days after challenge and RSV titers in **(B)** lungs and **(C)** nasal turbinate were determined via plaque assay. Data shown are mean RSV PFU per gram of tissue (pfu/g) and SD for 6–8 animals per group. Significance was evaluated by one-way ANOVA. LLOD, lower limit of detection; ULOD, upper limit of detection; ns not significant.

Weight-based doses of MEDI8897* and MEDI8897*-TM were administered, followed by an RSV A2 challenge (**Figure 3A**). RSV titers were determined in the lung and nasal turbinate on Day 4 post challenge. Prophylactic administration of MEDI8897* or MEDI8897*-TM resulted in a dose-dependent reduction in RSV replication in the lungs (**Figure 3B**) and nasal turbinate (**Figure 3C**) of infected cotton rats. As expected, the non-specific R347 control did not restrict viral replication following challenge. The loss of Fc effector function had no effect on viral titer reduction, with no statistically significant difference in RSV titers observed between MEDI8897* and MEDI8897*-TM across each dose level in the lungs and the nasal turbinate (ns, p>0.05).

## 4 Discussion

The direct provision of neutralizing mAbs is an appealing strategy for alleviating the severity of pediatric RSV disease while overcoming the limitations of the developing immune system (8, 44). Nirsevimab and palivizumab have established efficacy in protecting young infants against medically attended RSV LRTI, which is believed to be primarily dependent on direct virus neutralization (12, 17–19, 23–25, 45). A growing body of evidence suggests that broader antibody Fc effector functions are important mediators of RSV disease severity (31–33). However, Fc effector functions have also been linked to RSV symptom exacerbation and ADE in previous studies of maternally transferred antibodies and FI-RSV vaccinees (33, 46, 47). In this analysis, we used a combination of preclinical and *ex vivo* serological models, demonstrating that nirsevimab and palivizumab possess comparable levels of Fc effector activity and direct virus neutralization is the primary mechanism of nirsevimab-mediated protection.

A previous report by Hiatt, et al. illustrated that palivizumab’s ability to mediate Fc effector functions inversely correlated with RSV lung titers in the cotton rat model, suggesting that palivizumab protection could be attributed to a combination of virus neutralization and Fc effector activities (35). FcγRs are broadly classified into activating or inhibitory receptors based on the presence of an immunoreceptor tyrosine activating motif or immunoreceptor tyrosine inhibitory motif in the intracellular domain (48). SPR data generated in this analysis demonstrate that palivizumab and MEDI8897* display comparable binding affinities for a range of activating (FcγRI, FcγRIIA, and FcγRIIIA-158V) and inhibitory (FcγRIIB) FcγRs. We observed a modest reduction in FcγR binding affinity for nirsevimab compared with that for MEDI8897*, suggesting that the YTE modification results in a slightly reduced FcγR binding affinity for nirsevimab. Additionally, the L234F/L235E/P331S TM modification ablated FcγR binding for MEDI8897*-TM, consistent with previous reports (37). These data indicate that MEDI8897* and nirsevimab can bind to both activating and inhibitory FcγRs, and thus should possess the ability to mediate Fc effector activity in preclinical and *ex vivo* models.

Nirsevimab and palivizumab exhibited similar levels of *in vitro* ADNP and ADCP activity. Neutrophils are an initial line of host defense against many viral infections; however, their precise role during RSV pathogenesis has been difficult to define (49, 50). Neutrophil infiltration into the respiratory tract is a characteristic feature of early RSV infection in hospitalized infants and infants with mild disease (51, 52). Neutrophil levels have been associated with peak RSV symptom severity and viral load, incriminating neutrophilic inflammation as a harmful response to RSV LRTI. Conversely, neutrophils do appear to possess protective anti-viral functions for optimal RSV immunity. For example, defective activation of neutrophils via toll-like receptor (TLR) 4 and reduced expression of TLR4 on airway neutrophils has been associated with severe RSV disease, and administration of neutrophil cathelicidin LL37 has been associated with the induction of type III interferons and lower viral loads in murine models following RSV challenge (50). Protective ADNP has been observed in humans following vaccination with the investigational adenovirus-vectored RSV vaccine, ad26.RSV-preF (31); vaccinees displayed increased levels of neutrophil phagocytosis compared with unvaccinated participants following RSV challenge. Additional analyses by the same authors demonstrated that a version of palivizumab with enhanced ADNP functionality (SDIEALGA-modified: G236A/S239D/A330L/I332E) was associated with increased protection in murine models following RSV challenge. ADCP was also associated with protection from RSV challenge in ad26.RSV-preF vaccinees (31). Although the Fc-mediated interactions of RSV-antibody immune complexes are an underexplored mechanism of RSV protection, phagocytosis by antigen presenting cells is fundamental to the generation of adaptive immune responses. Insights from murine models suggest that macrophages, rather than neutrophils, are necessary for antibody-mediated restriction of RSV infection; thus, ADCP warrants further investigation as a mechanism of RSV protection *in vivo* (53).

Nirsevimab and palivizumab also demonstrated similar levels of *in vitro* ADCD activity. Studies of complement-deficient mice have suggested that complement supports anti-RSV immune responses by directly supporting antibody-virus binding, opsonization of virally infected cells, and augmenting CD4+ T-cell responses (33). Although nirsevimab demonstrated ADCD activity in these *in vitro* and *ex vivo* models, we do not anticipate complement-dependent cytotoxicity (CDC) following nirsevimab administration *in vivo*. CDC is initiated following antibody-antigen binding on cells, by the formation of hexametric antibody structures through noncovalent Fc-Fc interactions, which recruit and activate C1 to trigger the complement cascade (54). Previous data by Booth, et al. have illustrated C1q binding by YTE-modified antibodies without development of CDC; these authors hypothesize that YTE-modified antibodies do not promote CDC because the neonatal FcγR binding residues involved in the formation of these antibody hexamers partially overlap with the location of the YTE modification (55).

Multiple immune effector cells have demonstrated ADCC *in vitro;* however, natural killer (NK) cells are understood to be the most important contributors to ADCC *in vivo* and initiate the killing of virally infected cells following engagement of FcγRIIIA (33). Reduced numbers of NK cells have been reported in infants with severe RSV infection, despite the presence of maternally transferred antibodies, suggesting that ADCC is particularly important for protection against RSV (56).

In this report, we explored anti-RSV mAb binding to FcγRIIIA in Jurkat cells expressing FcγRIIIA-158V, a polymorphism that has previously been demonstrated to increase NK cell affinity for IgG binding (57). We observed lower levels of *in vitro* ADCC activity with nirsevimab compared with palivizumab. We hypothesize that this minor decrease in activity may be due to the lower FcγRIIIA-158V binding affinity we observed for nirsevimab compared with palivizumab. NK cell ADCC is driven solely by the FcγRIIIA receptor. In contrast to this, ADNP is mediated by FcγRI and FcγRIIA, ADCP is controlled by FcγRI, FcγRIIA, and FcγRIIIA, while ADCD is initiated by complement components binding to IgG hexamers at the cell surface rather than engagement with FcγRs (48, 54). Thus, as the other Fc effector functions explored in these assays utilize multiple FcγRs, they may be less sensitive to differences in mAb binding affinities compared with ADCC. A possible explanation for the difference in FcγRIIIA-158V binding affinities between nirsevimab and palivizumab is the presence of the YTE modification, as similar levels of FcγRIIIA-158V binding were observed between MEDI8897* and palivizumab. Although comparatively lower than palivizumab, we note that nirsevimab ADCC activity was still significantly above background levels.

Nirsevimab administration increased *ex vivo* ADNP, ADCP, and ADCD activity compared with placebo in participant serum from the MELODY study (19). Notably, nirsevimab immunization did not lead to an increase in ADCC activity compared with placebo in these analyses. As nirsevimab Day 1, Day 15, and placebo samples displayed ADCC activity above that of IgG-depleted serum, we hypothesize that this similarity in ADCC activity may be attributed to the presence of maternally transferred anti-RSV pre-F protein antibodies, which have been previously observed to possess ADCC activity and persist in infant serum for 3–6 months after birth (34, 58–61). Consistent with the primary analysis, 90% (n = 36/40) of the MELODY participants who provided serum samples for this analysis were <6 months of age at Baseline, and thus, due to their age may have possessed high levels of maternal anti-RSV pre-F protein antibodies (19). As previously discussed, the other Fc effector functions described in this report utilize multiple FcγRs, while ADCC is mediated solely by FcγRIIIA binding. Thus, we propose that high levels of maternally transferred antibodies may mask any additional benefit of nirsevimab administration in these assays through competition for FcγRIIIA.

Nirsevimab, MEDI8897*, and MEDI8897*-TM exhibited similar neutralization activity against recombinant RSV viruses *in vitro*, suggesting that the YTE and TM modifications do not impact neutralization potency. As expected from previous analyses, MEDI8897*-derived antibodies exhibited greater neutralization potency compared with palivizumab (17). These data are important to consider alongside the findings of the cotton rat challenge experiments in which MEDI8897* and MEDI8897*-TM demonstrated an equivalent dose-dependent reduction in lung and nasal turbinate RSV titers and previous reports in which palivizumab failed to restrict RSV replication in nasal turbinates (17). Taken together, these data would suggest that while Fc effector functions can complement the protective capabilities of nirsevimab and palivizumab, their primary *in vivo* mechanism of protection is achieved via direct virus neutralization.

Exploratory serological analyses from pivotal nirsevimab studies demonstrate that a single injection of nirsevimab provides sustained high levels of anti-RSV pre-F protein neutralizing antibodies throughout an infant’s first RSV season, which prevents severe disease without impairing the development of natural antibodies against other RSV antigens (21). The high neutralizing potency of nirsevimab has been associated with minimal viral escape and neutralization of >99% of RSV variants observed during pivotal nirsevimab studies and variants in global circulation between 2015–2021 (21, 42). Post-mortem case reports of Baseline-seronegative vaccinees following FI-RSV vaccination suggest that the presence of poorly neutralizing immune complexes is one of several probable etiological causes of severe ADE (46). This hypothesis is supported by observations of abrogated ADE in complement C3-deficient mice following FI-RSV vaccination, which suggest that poorly neutralizing immune complexes retained FcγR activity and contributed to ADE (46).

These observations with FI-RSV antibodies are important to consider in the context of the safety observed with therapeutic RSV mAbs (11, 62, 63). Palivizumab has a well-documented history as an effective form of RSV immunoprophylaxis with a manageable safety profile throughout its >25 years of use (11, 12). Although palivizumab use has declined following the recommendation of the American Academy of Pediatrics in 2014 to reserve its use solely for infants at high risk of severe RSV disease, palivizumab continues to be administered to thousands of high-risk infants on a monthly basis throughout a typical RSV season without any safety concerns (11, 64). In contrast to the poorly avid antibodies induced by FI-RSV, palivizumab has been observed to possess high levels of neutralizing activity and thus forms potently neutralizing immune complexes against a large proportion of historical and contemporary RSV viruses (17, 21). Importantly, no cases of ADE have been associated with palivizumab use, despite documented palivizumab Fc effector activity (31, 35). In this analysis and in previous reports, we have shown that nirsevimab possesses further neutralizing potency beyond that of palivizumab, and thus, is likely to protect against severe RSV disease without incurring increased risk of ADE (17).

Limitations of these analyses include the use of pooled MELODY participant serum rather than individual participant data, which did not allow for analysis of variation in Fc effector activity between individual participants in the *ex vivo* analyses. Additionally, the Day 15 serum was collected from MELODY participants enrolled at sites within the European Union and thus constitutes only a subset of the overall enrollment cohort (nirsevimab: 1.5% [n = 30/2009]; placebo: 1.0% [n = 10/1003]). Our results represent the nirsevimab Fc effector activity in serum shown at Day 15 rather than through to Day 151. Although this allowed us to assess early nirsevimab activity, we are unable to infer if this is maintained throughout the established efficacious period for nirsevimab (>150 days post dose). The cotton rat model used for our *in vivo* analyses of MEDI8897* and MEDI8897*-TM was also not thoroughly representative of human disease, and higher concentrations of nirsevimab are required for optimal protection in humans (18, 19, 23–25, 65). We acknowledge that widespread use of nirsevimab may impose a selection pressure on circulating RSVs, which could theoretically lead to treatment-emergent viral variants with reduced mAb binding capabilities. It is possible that Fc-mediated effector functions of nirsevimab will remain against viruses that have substitutions responsible for escape of nirsevimab-mediated neutralization activity. However, we have not evaluated the relative importance of nirsevimab direct virus neutralization and Fc effector-mediated activities for protection against potential RSV variants with reduced mAb binding. Importantly, nirsevimab has retained its neutralization activity against >99% of RSVs observed in pivotal clinical studies and among circulating viruses since 2015, and further viral surveillance studies alongside nirsevimab rollout are planned (21, 42). Despite the limitations of these analyses, we believe our data provide a comprehensive insight into the Fc effector functions of nirsevimab and provide valuable evidence on the mechanisms of nirsevimab-mediated protection.

In conclusion, our data demonstrate that nirsevimab possesses comparative Fc effector activity to that of palivizumab and confirm that direct virus neutralization is the primary mechanism of nirsevimab-mediated protection. These findings complement existing clinical data illustrating reduced RSV LRTI following nirsevimab administration during pivotal clinical and real-world evidence studies, and collectively support the use of nirsevimab for the benefit of neonates and infants in their first RSV season, and in medically vulnerable children up to 24 months of age who remain vulnerable to severe RSV disease in their second RSV season (18, 19, 23–25, 66).

## Data availability statement

Data underlying the findings described in this manuscript may be obtained in accordance with AstraZeneca’s data sharing policy described at https://astrazenecagrouptrials.pharmacm.com/ST/Submission/Disclosure. Data for studies directly listed on Vivli can be requested through Vivli at www.vivli.org. Data for studies not listed on Vivli could be requested through Vivli at https://vivli.org/members/enquiries-about-studies-not-listed-on-the-vivli-platform/. AstraZeneca Vivli member page is also available outlining further details: https://vivli.org/ourmember/astrazeneca/. Further inquiries can be directed to the corresponding authors.

## Ethics statement

All procedures were performed in accordance with federal, state, and institutional guidelines in an AAALAC-accredited facility; the MedImmune IACUC board approved this research under a specified protocol (MI-16-0014); and all animal work was performed in accordance with the IACUC policies. The regulations achieve the standard of care required by the US Department of Health and Human Services’ Guide for the Care and Use of Laboratory Animals. Animal studies were conducted according to Good Laboratory Practice regulations for nonclinical laboratory studies. The study was conducted in accordance with the local legislation and institutional requirements. The MELODY trial was performed in accordance with the principles of the Declaration of Helsinki and the International Council for Harmonisation Good Clinical Practice guidelines. Each site had approval from an institutional ethics review board or ethics committee, and appropriate written informed consent was obtained for each participant.

## Author contributions

TB: Conceptualization, Data curation, Formal Analysis, Investigation, Methodology, Writing – review & editing, Project administration, Visualization. CC: Formal Analysis, Investigation, Methodology, Writing – review & editing, Supervision. TR: Formal Analysis, Methodology, Writing – review & editing, Data curation, Resources, Visualization. SS: Data curation, Formal Analysis, Methodology, Visualization, Writing – review & editing, Investigation, Validation. HJ: Formal Analysis, Investigation, Methodology, Writing – review & editing. LM: Methodology, Writing – review & editing. TZ: Writing – review & editing, Formal Analysis. DW: Writing – review & editing, Conceptualization, Data curation, Project administration, Visualization. KT: Conceptualization, Data curation, Writing – review & editing, Formal Analysis, Investigation, Methodology, Supervision. EK: Conceptualization, Methodology, Project administration, Resources, Supervision, Writing – review & editing.
